# Uncommon functional properties of the first piscine 26S proteasome from the Antarctic notothenioid *Trematomus bernacchii*

**DOI:** 10.1042/BSR20160022

**Published:** 2016-04-15

**Authors:** Marta Gogliettino, Marco Balestrieri, Alessia Riccio, Angelo Facchiano, Carmela Fusco, Vincenzo Cecere Palazzo, Mosè Rossi, Ennio Cocca, Gianna Palmieri

**Affiliations:** * Institute of Biosciences and BioResources, National Research Council (CNR-IBBR), Via Pietro Castellino 111, 80131 Napoli, Italy; † Institute of Food Sciences, National Research Council (CNR-ISA), Via Roma 64, 83100 Avellino, Italy

**Keywords:** 26S proteasome, Antarctic notothenioid *Trematomus bernacchii*, cold adaptation, oxidized protein degradation, protein degradation machinery

## Abstract

Protein homoeostasis is a fundamental process allowing the preservation of functional proteins and it has a great impact on the life of the Antarctic organisms. However, the effect of low temperatures on protein turnover is poorly understood and the cold-adaptation of the degradation machinery remains an unresolved issue. As the 26S proteasome represents the main proteolytic system devoted to the controlled degradation of intracellular proteins, the purpose of the present study was to investigate the functions of this complex in the notothenioid *Trematomus bernacchii*, in order to better understand its role in the physiology of Antarctic fish. To this aim, we purified and characterized the 26S proteasome from *T. bernacchii* and isolated the cDNAs codifying seven of the 14 subunits belonging to the proteasome 20S core particle. Results provided evidences of the high resistance of the piscine 26S proteasome to oxidative agents and of its ‘uncommon’ ability to efficiently hydrolyse oxidized bovine serum albumin (BSA), suggesting that this enzymatic complex could play a key role in the antioxidant defense systems in fish inhabiting permanently cold marine environments. These unique properties were also reflected by the 3D model analysis, which revealed a higher structural stability of the piscine complex respect to the murine template. Finally, a comparative analysis, performed in a variety of tissues collected from *T. bernacchii* and the temperate fish *Dicentrarchus labrax*, showed a lower protein retention in the cold-adapted fish, possibly due to a better efficiency of its degradation machinery.

## Abstract (precis)

The antioxidant defense mechanisms have a great impact on the life of Antarctic organisms. The present study could represent the first evidence of a direct involvement of the 26S proteasome in the antioxidant defense systems of fish adapted to cold.

## INTRODUCTION

In the last decades, several studies have been focused on the strategies adopted by polar fish to counteract the extreme temperature conditions of inhabiting sub-zero environments. Evolution in the Antarctic marine organisms has resulted in a series of physiological and biochemical adaptations that include antifreeze protein production, elevated blood osmotic concentrations, mitochondrial proliferation and thermal compensation of metabolic activity [1–4].

Protein homoeostasis represents a fundamental process which allows the preservation of all the functional cellular proteins and it has a great impact on the life of marine Antarctic habitats [5]. However, managing of cold-related protein damage and adaptation of the protein degradation machinery in polar organisms remain a yet unresolved issue in the evolutionary biology.

Previous research on Antarctic notothenioids revealed the absence in these species of defense induction mechanisms represented by the heat shock response [6], thus suggesting that this restoring protein function has been lost, possibly due to the lack of a positive selection during evolution at stable sub-zero temperatures [6]. Furthermore, several studies have reported the *in vitro* denaturing effects of cold temperatures on 3D structures of proteins, although it is still unclear whether this is also reflected into higher levels of damaged proteins *in vivo* [7–10]. Low temperatures alter either the rate of protein synthesis than the protein folding, resulting in *not-functional* conformations and an imbalance in the synthesis and degradation processes [2,3]. Therefore, a constant and dynamic regulation of these pathways is essential for cell viability in all living organisms as it is essential to elude the accumulation of damaged proteins that induce cytotoxic effects. The ubiquitin (Ub)–proteasome system (UPS) represents the main pathway responsible for breaking down proteins, which involves two successive steps: (1) tagging of the substrate by covalent attachment of multiple Ub molecules, and (2) degradation of the tagged protein into small peptides by 26S proteasome with release of free and reusable Ub molecules [11,12]. The 26S proteasome is a giant machine containing a proteolytic core particle 20S (CP) capped at one or both ends by a 19S regulatory particle (RP). The CP is a barrel-shaped complex of 28 subunits (alpha and beta) that harbours three distinct proteolytic active sites (beta 1, beta 2 and beta 5), in the two central beta-rings [13–16]. Information on UPS in fish species is generally scarce and few data on the regulation of protein degradation processes specifically in Antarctic fish, are available [5,10,17].

Notothenioids represent 55% of the fish species in the Southern Ocean and in many coastal shelf areas they represent over 90% of the fish biomass [3,18]. A previous investigation on Ub-conjugated protein levels, which have been considered a good indicator of the cellular protein integrity, provided evidences that the low temperatures of the Antarctic marine environments can significantly affect the protein functions [10], although more studies are still necessary to better understand the physiological constraints of maintaining protein homoeostasis in these polar species. Accordingly, in the last years, there has been a growing interest in studying the oxidative stress phenomena in fish inhabiting Antarctic oceans [19–21], which should have developed sophisticated antioxidant defense systems to counteract the side effects of life at low temperatures [17,20–22]. In mammals, a large body of evidences demonstrated that the 20S proteasome is the main proteolytic factor able to efficiently remove oxidatively damaged proteins [13–16,23]. In contrast, 26S proteasome turns out to be very poor at degrading these molecular species and there are no clear indications on the involvement of this enzymatic complex in antioxidant defense systems in eukaryotic organisms [24–26].

The present study was undertaken to investigate the proteasomes from the notothenioid *Trematomus bernacchii*, which belongs to the endemic class of fish in Antarctic waters, living at temperatures below 1.5°C. To this aim, we purified and characterized the piscine 26S proteasome and isolated the cDNAs codifying seven of 14 subunits of the 20S complex, in order to understand its role in the physiology of a red-blooded Antarctic fish. Our data demonstrated that the piscine 26S proteasome was highly resistant to oxidative agents and able to efficiently hydrolyse oxidized bovine serum albumin (BSA), unlike the mammalian counterparts [24–26], suggesting that it could play a key role in the antioxidant defense systems. Unique properties were also found by the 3D models analysis, which revealed a higher structural stability of the piscine complex respect to the murine template. Furthermore, a lower Ub-protein level was detected in a variety of *T. bernacchii* tissues respect to that observed in the temperate fish *Dicentrarchus labrax*, possibly due to a more efficient degradation machinery and/or a reduced ubiquitination process. Therefore, our results provided the first evidence on the role of the 26S proteasome in the protein homoeostasis in a polar fish, suggesting that the cold adaptation could have stimulated an improvement in the molecular recognition events of the UPS pathway.

## MATERIALS AND METHODS

### Ethical procedures

The sample collection and experimental research conducted on the animals utilized in the present study were according to the law on activities and environmental protection in Antarctica approved by the Ministry of Foreign Affairs of the Republic of Italy (MAE), to comply with the ‘Protocol on Environmental Protection to the Antarctic Treaty’, Annex II, art.3. All procedures, including euthanasia, were reviewed and approved by MAE and performed in accordance with the European Communities Council Directive of 24 November 1986 (86/609/EEC).

### Animal sampling

Specimens of *T. bernacchii* were fished in the vicinity of Mario Zucchelli Station, along the coast of Terra Nova Bay (74′42°S, 164′07°E), Antarctica, during the Italian XXVII and XXIX expeditions (December 2011 to January 2012 and January to February 2014 respectively). They were maintained in running seawater at -2°C to +1°C until tissue sampling. *D. labrax* specimens were collected at a fish farm, where the water temperature was maintained at 18°C. The animals were anesthetized with tricaine methanesulfonate (MS222, 300 mg/l) for at least 30 min before being killed by truncation of the spinal cord. Tissues were dissected from adult specimens, and frozen immediately in liquid nitrogen. Blood was drawn from the caudal vein with heparinized syringes. Blood cells were collected by centrifugation at 3000 ***g*** for 5 min, washed in 1.7% NaCl, and then frozen in liquid nitrogen. Tissues and cells were stored at -80°C until use.

### Proteasome activity and Ub-protein levels

*T. bernacchii* and *D. labrax* hemolysates were obtained from erythrocytes by incubation in hypotonic solution (25 mM Tris/HCl, pH 7.5) for 30 min on ice and centrifugation at 9200 ***g*** for 40 min at 4°C. Tissue samples were homogenized in four volumes of ice-cold homogenization buffer (10 mM Tris/HCl, pH 7.5, containing 150 mM NaCl) with an Ultra-Turrax T25 homogenizer (IKAWorks). The homogenates were centrifuged at 288000 ***g*** for 1.5 h at 4°C and the supernatant used for the next experiments. Total protein concentration of the tissue homogenates was determined using the Bradford protein assay [27] for normalization of sample protein content. Equal amounts of total proteins from each tissue were used for dot blot analysis by spotting the samples through circular templates directly on to the nitrocellulose membrane. Following blocking, the membrane was incubated with Ub conjugates specific primary antibody HRP conjugate (1:2500, produced by Enzo Life Sciences) for 1 h at room temperature. Enhanced chemiluminescence and autoradiography (Amersham Biosciences) were used to displayed immune complexes formed and measured by densitometry analysis with ChemiDoc XRS (Bio-Rad Laboratories). Chemiluminescence was quantified using Quantity One Software (Bio-Rad Laboratories) and the acquisition data were performed under conditions to prevent saturation of the chemiluminescence signal. The chymotrypsin-like (CT-like) proteasomal activity for each tissue under analysis was revealed using the fluorogenic substrate *N*-succinyl-Leu-Leu-Val-Tyr-7-amido-4-methylcoumarin (LLVY, purchased for Sigma–Aldrich). The assays were monitored in a Perkin–Elmer LS 50B fluorimeter. The excitation and emission wavelengths were 380 nm and 460 nm respectively. The *K*_m_ determination of proteasome using LLVY as substrate was performed on pancreas, heart, muscle and trunk kidney protein extracts from *T. bernacchii* and *D. labrax,* after one purification step on Superose 6 gel filtration column following the procedure described below.

### 26S proteasome preparation

The proteasome active fractions, recovered after each purification step, were detected by measuring the CT-like activity using the specific fluorogenic substrate LLVY. The *T. bernacchii* hemolysates were loaded on a DEAE Sepharose Fast Flow column, previously equilibrated in 25 mM Tris/HCl, pH 7.5 (Buffer A), and connected to an AKTA_FPLC_ system (Amersham Biosciences). Bound proteins were eluted using an ionic strength gradient from 0 to 1 M NaCl in Buffer A at a flow rate of 1 ml·min^−1^. The active fractions were pooled, dialysed extensively against 25 mM Tris/HCl, pH 7.5 and 1 M ammonium sulfate (Buffer A) and then loaded on to a Phenyl Sepharose (Amersham) column, connected to an AKTA_FPLC_ system (Amersham Biosciences), equilibrated in the same buffer. Bound proteins were eluted with a linear gradient (0–100%) of 25 mM Tris/HCl (pH 7.5) (buffer B) at a flow rate of 1 ml·min^−1^. The active fractions were pooled, dialysed against 25 mM Tris/HCl (pH 7.5) and then applied to a Superdex 200 PC 3.2/30 column connected to a SMART System (Pharmacia), equilibrated in 25 mM Tris/HCl, pH 7.5 and 50 mM NaCl. Finally, active fractions were pooled and the purified proteasome was stored in 25 mM Tris/HCl pH 7.5, containing 5% glycerol.

### Molecular mass determination

Molecular mass of the native 26S proteasome was established by gel filtration chromatography on Superdex 200 and Superose 6 PC 3.2/30 columns (Pharmacia Biotech), connected to a SMART System, equilibrated in 25 mM Tris/HCl, pH 7.5 and 50 mM NaCl, and calibrated with molecular mass standards (26S human proteasome 2100 kDa, 20S human proteasome 700 kDa, Apoferritin 443 kDa, porcine Acylpeptide hydrolase 300 kDa, bovine serum albumin 66.5 kDa, chymotrypsin 25 kDa). The protein concentration was determined with the Bradford assay method [27].

### Enzyme assays

Enzyme assays were performed by spectroscopic fluorescence, using the typical substrates for the detection of the different proteasome activities: LLVY for CT-like activity, *tert*-butyloxycarbonyl-Leu-Arg-Arg-7-amido-4-methylcoumarin (LRR, purchased from Boston Biochem) for trypsin-like (T-like) activity and *tert*-butyloxycarbonyl-Leu-Leu-Glu-7-amido-4-methylcoumarin (LLE, purchased from Sigma–Aldrich) for caspase-like (PGPH-like) activity. The release of fluorescent product [7-amino-4-methylcoumarin (AMC)] was monitored at 380 nm and 460 nm, as excitation and emission wavelengths respectively, using a Jasco FP-8200 spectrofluorometer, equipped with a thermostated cuvette compartment. All experiments were carried out in triplicate on three different protein preparations. The reaction mixture (0.8 ml), containing the appropriate amount of enzyme in 50 mM Tris/HCl buffer at optimal pH, temperature and SDS concentrations, was preincubated for 5 min. Then, the specific substrate was added and the release of product was measured. Calculated activities were based on the initial linear phase of release and all the enzymatic activities were expressed in arbitrary units.

### pH, temperature and SDS effects on proteasome activities

For all assays, activities were measured as described above using LLVY, LRR and LLE as substrates. Effect of pH was determined between pH 5.0 and 10.0. Sodium acetate buffer (50 mM) was used for pH values ranging from 5.0 to 6.0, replaced by Tris/HCl (50 mM) buffer in the 6.5–9.0 pH range and by CAPS at pH 10.0. Temperature effect was analysed between 15 and 60°C. Relative activity was expressed as a percentage of the maximum of the enzyme activities under the standard assay conditions. The thermal stability was determined by measuring residual activities after incubation of the enzyme at various temperatures (10°C; 37°C; 45°C). Temperatures below 10°C were not explored due to their dramatic influence on substrate solubility. For the SDS effect, the proteasome was preincubated with the detergent (0–0.2%) at 37°C before addition of the adequate substrate. The relative activity was expressed as percentage of the activity respect to the control (0% SDS) under the standard assay conditions.

### *K*_m_ determination

The temperature–*K*_m_ effects were determined at different temperatures using LLVY as substrate. All experiments were carried out in triplicate on two different protein preparations. Data were fitted to the Michaelis–Menten equation by a nonlinear regression with the GraphPad Prism software.

### Western blot analysis

Samples were run on SDS/PAGE (12%) and then electroblotted on to PVDF membranes (ImmobilonTM, Millipore). Membranes were next incubated with the piscine anti-beta 1/beta 5 (LS-C111925 rabbit IgG, 1:1000, purchased from LifeSpan BioSciences) and piscine anti-Rpt1 (LS-C290473 rabbit IgG, 1:1000, purchased from LifeSpan BioSciences) primary antibodies (1 h at room temperature) and then with the HRP-conjugated secondary antibodies (1 h at room temperature). Immune complexes formed were visualized by enhanced chemiluminescence and autoradiography (Amersham Biosciences) and measured by densitometry analysis with ChemiDoc XRS (Bio-Rad Laboratories). Protein expression data were quantified with Quantity One Software (Bio-Rad Laboratories).

### Gel electrophoresis

SDS/PAGE was carried out according to the procedure described by Laemmli [28]. Standard proteins (Broad Range) were from New England BioLabs. Polyacrylamide gel electrophoresis under non-denaturing conditions (Native-PAGE) was performed according to the method described by Holzl et al. [29]. Proteasome activity was detected by in-gel peptidase activity performed as previously reported [30] with some modifications. Specifically, the gel was immersed in 50 mM Tris/HCl, pH 9.0, 0.02% SDS and 100 μM LLVY at 37°C. The fluorescence was detected 30 min after exposure to the fluorogenic peptide. For immunoblotting, proteins in native gels were transferred to PVDF membranes following the same protocols described in Western blot analysis section.

### Degradation of oxidized BSA by proteasomes

BSA (purchased from Sigma–Aldrich) was used as a model of proteolytic substrate. The oxidized and unoxidized BSA were prepared as described by Fujino et al. [31]. The oxidant resistance of 26S proteasome complex was tested with H_2_O_2_. Exposure of the proteasome (8 μg) to different H_2_O_2_ concentrations (1, 3, 5, 10 and 50 μmol/mg protein) was carried out for 24 h at 37°C in 20 mM Tris buffer, pH 7.5 and the reaction mixtures were subjected to native-PAGE analysis. Solutions of unoxidized (3 μg) and oxidized BSA (10 μg) were incubated with untreated and H_2_O_2_-treated 26S proteasome (protein ratio of 200 μg substrate protein/7 μg of proteasome) in 30 μl of 50 mM Tris/HCl, pH 8.0 at 37°C for 2 and 24 h. Then, the reaction mixtures were subjected to SDS/PAGE analysis.

### Cloning

Total RNAs from several tissues of *T. bernacchii* were isolated according to the RNeasy Plus Universal Mini Kit (Qiagen) protocol. RNA concentrations were determined with a Qubit Fluorometer (Invitrogen). RNAs were then reverse transcribed with the SuperScript VILO MasterMix (Invitrogen). The cDNAs of the proteasome subunits under investigation were amplified by PCR with oligonucleotides designed on the homologues sequences from *Gasterosteus aculeatus*, *Oreochromis niloticus*, *Xiphophorus maculatus, Takifugu rubripes* and *Danio rerio*, and on the BLAST analysis of several SRA libraries from Notothenioidei (SRX088548, SRX089044–9, SRX373094–100, SRX305406, SRX306432, SRX306459, SRX306462–4).

The oligonucleotides are listed in Table 1. The amplifications were performed as follows: 94°C for 2 min, 40 cycles of 94°C (30 s), 58–62°C (30 s) and 72°C (1 min), and a final extension at 72°C for 10 min. The PCR products were analysed on 1% agarose gel, purified with the StrataPrep DNA Gel Extraction Kit (Stratagene) and cloned into the StrataClone PCR Cloning kit (Stratagene).

**Table 1:** Primers used for the amplifications of the *T. bernacchii* proteasome subunits cDNAs

Primer	SEQUENCE	*T*_m_ (°C)
Alpha 4for	5′-GTAGTGGACCTCTTATTCTGTAGG-3′	58.4
Alpha 4rev	5′-CAATACAGGATTTGGTGACAGG-3′	58.1
Alpha 5for	5′-GACGCTTCCCCTGAAACAAG-3′	60.6
Alpha 5rev	5′-GTGACATTCAGCCCAGGTG-3′	60.1
Alpha 7for	5′-GGCTTCACAAATTGCTAACTAGC-3′	59.5
Alpha 7rev	5′-CTCAAGTTATGATTTAGCTCTGCACA-3′	60.2
Beta 1for	5′-CCATATTGCAGTGATACAGCGAG-3′	60.5
Beta 1rev	5′-CTCAGTCCTTCCTCAGGGG-3′	60.1
Beta 2for	5′-GTCGGGATACAGGGACCG-3′	60.8
Beta 2rev	5′-AGCGGTCACTTGGCGC-3′	62.8
Beta 3for	5′-CACAATAGCCAAGAAAAAGTGGAG-3′	59.3
Beta 3rev	5′-GTTCGTGTGGTGATCTTGTCC-3′	60.4
Beta 5for	5′-GGGAGTTTCAAAGATGGCTCT-3′	59.2
Beta 5rev	5′-TTGTACTGCTGGTGCAGCAT-3′	61.7

### Sequence analysis

The sequences of the seven cloned cDNAs were determined with an ABI PRISM 3100 automated sequencer at PRIMM. The sequences were edited and analysed with the CLC Main Workbench 7.6 program (CLC bio, 2015) and deposited in the GenBank database under the accession numbers KP735942 (*beta 1_Tb_*), KP735943 (*beta 2_Tb_*), KP735944 (*beta 5_Tb_*), KP735945 (*alpha 4_Tb_*), KP735946 (*alpha 5_Tb_*), KP735947 (*alpha 7_Tb_*) and KP735948 (*beta 3_Tb_*).

The amino acid sequences of the *T. bernacchii* proteasome subunits were aligned with their homologues retrieved from databanks by use of the MUSCLE program.

### Phylogenetic analysis

Phylogenetic and molecular evolutionary analyses for the three catalytic proteasome subunits (beta 1, beta 2 and beta 5) were carried out using MEGA version 6 [32]. The alignment containing all the amino acid sequences belonging to these three subtypes, together with the sequence of the proteasome beta subunit of the archaebacterium *Thermoplasma acidophilum* used as outgroup, was tested by ‘Find Best DNA/Protein Models (ML)’ option to search the most appropriate evolutionary model. Maximum likelihood analysis was performed with the ‘Construct/Test Maximum Likelihood Tree (ML)’ option, using the parameters indicated by the evolutionary model (substitution model LG+G, gamma=3.7136) and the bootstrap test with 1000 replicates. Finally, a phylogenetic tree with the best bootstrap consensus was obtained [32].

### Molecular modelling

Molecular modelling of the 20S seven protein subunits was performed in agreement with well established procedures applied in our labs [33]. The search for templates were performed by using BLAST at the NCBI web site. The best template structures were found belonging to the mouse proteasome (PDB code 3UNB). The subunits have been modelled as single chain, according to the related chain in 3UNB: alpha 4, chain B; alpha 5, chain D; alpha 7, chain C; beta 1, chain L; beta 2, chain J; beta 3, chain I; beta 5, chain K. The percentage of sequence identity was in all cases >80%, thus making very simple the correct alignment of the target and template sequences. Modeller 9.12 [34] was used to create 10 models for each chain, and the best was selected on the basis of the model energy, evaluated by PROSA-web server [35] and the backbone stereochemistry, evaluated by PROCHECK software [36]. Models were also analysed for the presence of H-bonds by HBplus [37] and salt-bridges, by using an original software that apply criteria similar to the online tool described by Paladino et al. [38] with specific filters for simplify the finding of intra- and inter-chain interactions. Presence and conservation of cysteines and amino acids involved in salt bridges were checked manually on the multiple alignments.

## RESULTS AND DISCUSSION

### Tissue levels of ubiquitinated proteins and proteasome activity in *T. bernacchii* and *D. labrax*

**Figure 1:**
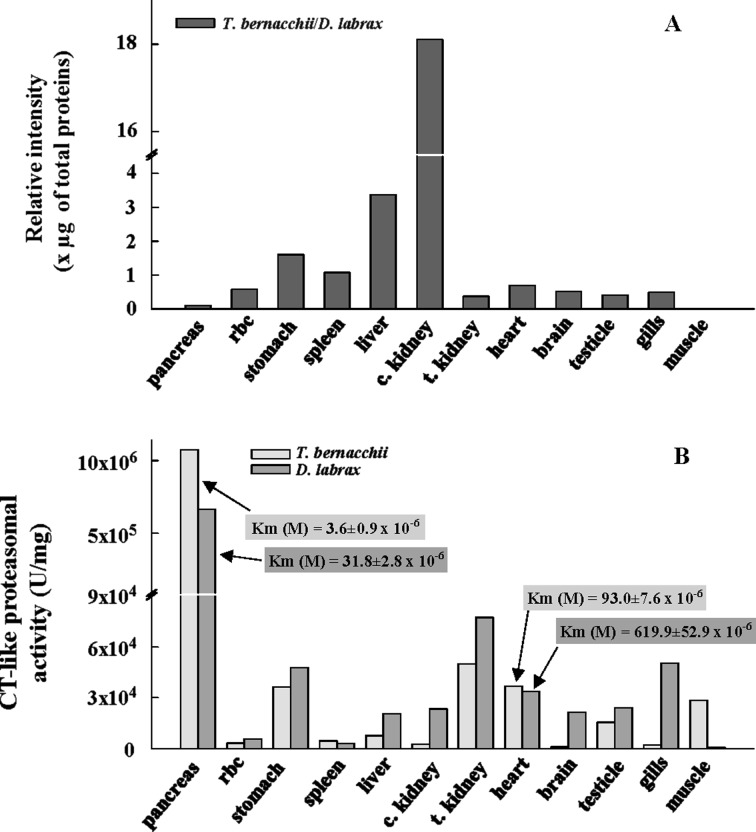
Analysis of Ub-conjugated protein levels and CT-like proteasomal activity in different tissues of *T. bernacchii* and *D. labrax* (**A**) Ub-conjugated protein levels in different tissues of *T. bernacchii* and *D. labrax* determined by Dot Blot densitometric analysis. Ubiquitin-conjugated protein levels are reported as relative intensity determined by comparing the dot blot values of the *T. bernacchii* tissues respect to the corresponding tissues of *D. labrax*. (**B**) CT-like proteasomal activity (beta 5 subunit) measured using LLVY as substrate and expressed in arbitrary units. The enzymatic units (U) corresponded to the maximal activity (*V*_max_) measured in each tissue protein extract. Data were expressed as mean from experiments performed in triplicates on three different protein preparations and all standard deviation values were lower than 5%.

Protein degradation is a critical determinant of growth and it is influenced by the temperature in a fashion independent from that of protein synthesis [5,10]. In these cellular processes, UPS has emerged as a central player, but functional and structural information on this complex machinery in fish are still scarce. Therefore, to understand the effects of sub-zero environments on the accumulation of the ubiquitinated (Ub)-proteins, we compared the levels of these conjugates in a variety of tissues of the Antarctic fish *T. bernacchii* and the temperate species *D. labrax*. The same analysis was performed evaluating the total CT-like proteasome activity associated to the beta 5 catalytic subunit. Unexpectedly, the Ub-conjugated protein levels in *D. labrax*, quantified by Dot Blot densitometric analysis, were significantly higher than those detected in *T. bernacchii* in pancreas, trunk kidney, testicle, gills, muscle, heart and brain, as evidenced by the low ratio values (less than 1) between the corresponding intensities in these tissues (Figure 1A). On the contrary, *T. bernacchii* showed higher levels of Ub-conjugate proteins in liver and cephalic kidney (ratio values more than 1) and comparable amounts in the remaining analysed tissues respect to *D. labrax*. In addition, the total CT-like activity of proteasome in *D. labrax* was generally higher than that measured in *T. bernacchii* in all the tissues under investigation except for the muscle and pancreas (Figure 1B), suggesting a potentially more efficacious degradation machinery in the temperate species.

These results seemed to be in contrast with those observed analysing the substrate affinity values (*K*_m_) using the substrate LLVY, which is widely used for the detection of CT-like proteasome activity. The *K*_m_ values were only evaluated in pancreas, heart, muscle and trunk kidney, as the very complex protein extracts strongly affected this analysis. The substrate affinity of the proteasome appeared improved in *T. bernacchii* at least in pancreas and heart, possibly resulting in an increased efficiency of UPS pathway (Figure 1B). However, the *K*_m_ values were similar in muscle and trunk kidney (*K*_m_ values ranging from 80 to 100×10^−6^ M and 60–80×10^−6^ M respectively) as typically evidenced among the orthologous enzymes [2]. These results were also confirmed on partially purified proteasome preparations (see Materials and Methods).

The obtained data revealed, for the first time, increased Ub-protein levels in a temperate fish, that could derive from a reduced cellular capacity in the degradation of ubiquitinated proteins respect to that observed in a cold-adapted species. In this case, an optimization of the thermodynamic parameters of the catalytic proteasome subunits (at least the beta 5), which results in an improvement of molecular recognition events, could contribute to maintain a high protein degradation efficiency.

### Purification of proteasome from *T. bernacchii* erythrocytes

**Figure 2:**
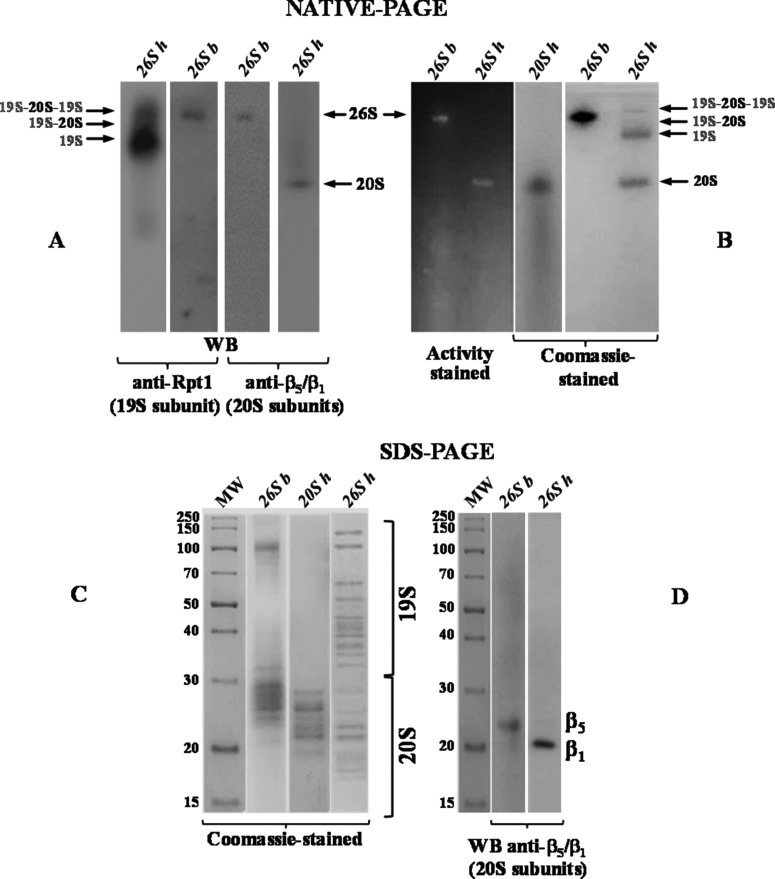
Native-PAGE, SDS/PAGE and Western blot analyses of the 26S proteasome from *T. bernacchii* (**A**) Native-PAGE of the purified *T. bernacchii* 26S (26S *b*), immunoblotted against 19S subunit Rpt1 or 20S subunits β_5_/β_1_. The commercially available 26S from human erythrocytes (26S *h*) was used as positive control. (**B**) Native-PAGE of 26S *b* followed either by in-gel detection of CT-like activity (β_5_ subunit), using the fluorogenic substrate LLVY, than Coomassie-blue stained. 26S *h* and 20S *h* were used as positive controls. The main proteasome species are indicated: 19S–20S–19S, doubly capped 26S; 19S–20S, singly capped 26S; 19S (regulatory complex) and 20S (catalytic core), free particles. (**C**) SDS/PAGE analysis of 26S *b* in comparison with 26S *h* and 20S *h* used as controls. (**D**) SDS/PAGE of 26S *b* immunoblotted against 20S subunits β_5_/β_1_. The 26S *h* was used as control. The results were representative of three independent experiments on three different protein preparations.

Since very little is known about the structural and functional properties of proteasome in fish, we first developed a strategy to isolate and characterize this enzymatic complex from *T. bernacchii* red blood cells (RBCs). Proteasome detection and enrichment at each purification step was followed by immunoblot analysis and measuring the proteolytic activity towards the fluorogenic substrate LLVY. Purification of the proteasome to homogeneity was ultimately achieved by sequential anion exchange, hydrophobic interaction and size exclusion chromatographies. During the hydrophobic step, the activity profile revealed the presence of two peaks (data not shown): peak 1, containing the most of proteolytic activity and peak 2, containing high molecular mass contaminating proteins and the previously characterized protease APEH (acylpeptide hydrolase) [20]. For these reasons, peak 1 fractions were pooled and subjected to the next purification step. The protein homogeneity and its identification as the 26S proteasome was confirmed by several analytical procedures: **(1)** gel filtration chromatography using two different size-exclusion columns, which revealed a molecular mass of about 1400 kDa for the purified holoenzyme, in agreement with those reported for the singly-capped 26S proteasome (19S–20S) eukaryal counterparts (data not shown) [13–15]; **(2)** native-PAGE, displaying a unique intermediate electrophoretic band (Coomassie blue-stained) between those observed for the human doubly- and singly-capped 26S used as positive control [39] (Figure 2); indeed, as reported, the 26S human isoform easily dissociates into the 20S catalytic core and the regulatory complex (19S) [15,16,40], in contrast with the piscine isoform, that appeared to be highly stable; **(3)** native-PAGE (Figure 2), followed by in-gel detection of CT-like activity, which revealed a single fluorescent signal for 26S piscine isoform, corresponding to the Coomassie blue-stained band, under the assay conditions described in Materials and Methods. The same analysis, performed on the human 26S isoform, allowed to detect only the activity band corresponding to the 20S isoform (Figure 2), in agreement with that reported for eukaryal proteasomes [39,30]; **(4)** SDS/PAGE analysis (Figure 2), showing a wide range of molecular masses, from 22 kDa to over 150 kDa, thus mirroring what was previously described for eukaryal 26S proteasomes [14,29,41]; **(5)** Western blot analysis, performed using anti-beta 1/beta 5 subunits; **(6)** native-PAGE followed by immunoblotting against Rpt1 (19S subunit) and beta 1/beta 5 (20S subunits), evidencing immunoreactive signals in correspondence to the Coomassie blue-stained bands (Figure 2).

The 26S proteasome purification protocol is reported in Supplementary Table S1. The isoform was purified approximately 6-fold, with an activity recovery of 0.1% and a CT-like specific activity of 37900 U/mg. In addition, T-like and caspase-like (PGPH-like) hydrolysing activities were determined as 94750 and 60160 U/mg respectively [the enzymatic units (U) corresponded to the maximal activity (*V*_max_) measured].

Interestingly, the standard procedure developed to purify the mammalian 20S proteasome, omitting the use of glycerol and ATP, was sufficient to purify the piscine 26S isoform and preserve its integrity, thus suggesting a remarkable structural stability of *T. bernacchii* holoenzyme. Indeed, the presence of these two compounds in the purification buffers usually prevents the dissociation of the 26S proteasome into 19S and 20S particles [30].

### SDS effect on 26S proteasome CT-, T- and PGPH-like activities

**Figure 3:**
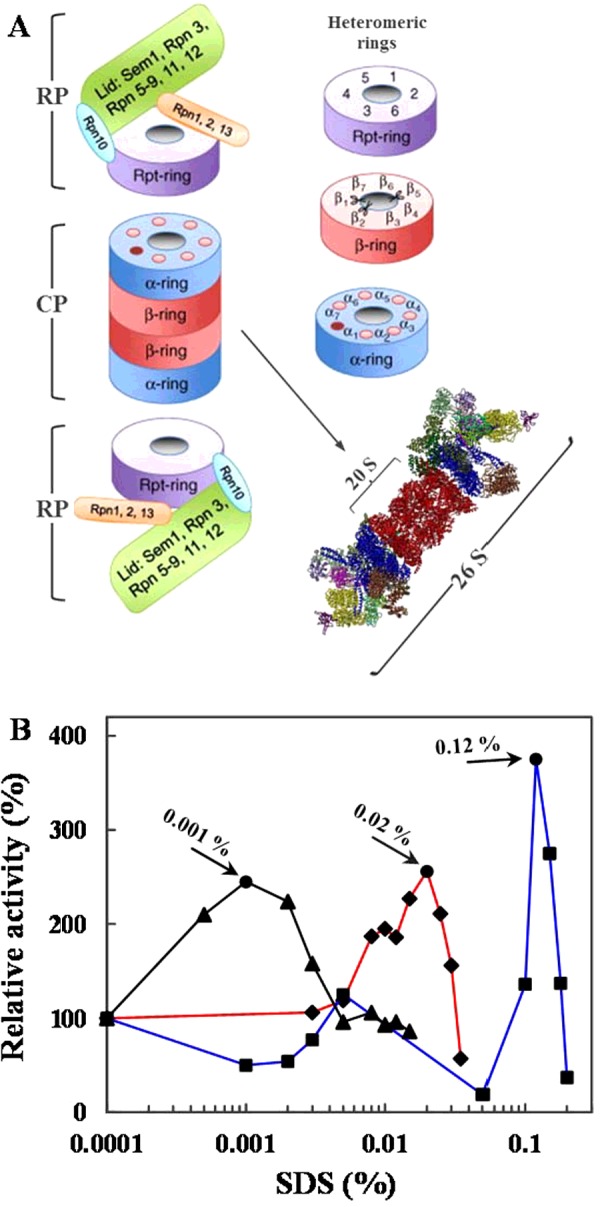
Effect of SDS on the proteasomal CT-like, T-like and PGPH-like activities (**A**) Schematic representation of the 26S proteasome. (**B**) The three different proteasome activities were measured after addition of increasing concentrations of SDS. The proteasome was preincubated 5 min with the detergent (0–0.2%) at 37°C before addition of the adequate substrate. The relative activity was expressed as percentage of the activity respect to the control (0% SDS). All experiments were performed in triplicate on three different protein preparations. T-like (beta 2), CT-like (beta 5) and PGPH-like (beta 1) activities are indicated in black, red and blue lines respectively.

As reported, the catalytic sites of mammalian 26S proteasomes are sequestered in the central hollow of its core particle 20S (Figure 3A) [14,15,42]. The entry into this chamber occurs via a narrow channel delimited by the outer alpha rings. Indeed, the N-terminus of the neighbouring alpha subunits maintains the 20S proteasome into an auto-inhibited state by an intricate lattice of interactions, hindering access to the channel (Figure 3A) [42]. Nevertheless, the exposure to a mild chaotropic agent, such as SDS, can stimulate the opening of the alpha-channel gate, allowing greater access of protein substrates [42–44]. As shown by SDS-mediated activation profiles reported in Figure 3(B), the effects of this denaturing compound on piscine 26S proteasome were dependent on the type of subunit considered. The T-like activity improved at the lowest SDS concentration tested (0.001%) and then restored to the original level with a further SDS increase. By contrast, CT-like and PGPH-like hydrolysing activities needed higher levels of SDS to reach their maxima. Specifically, the best CT-like activity was detected at 0.02% SDS, whereas the effect of this agent on PGPH-like activity was maximal at 0.12% SDS. Therefore, the activation of the three catalytic subunits were reached at very different SDS concentrations, in contrast with that described for the eukaryal 26S proteasomes [45,46]. In addition, it seems that the substrate used for T-like activity measurements was able to stimulate the gate opening without the contribution of high SDS concentrations, as already reported [45,46]. On the contrary, CT-like and PGPH-like activities needed increasing SDS amounts to allow the passage of the corresponding substrates by opening the entrance of the alpha-channel. Specifically, it appears that the optimal PGPH-like activity requires the maximum gate opening, which is triggered by an unusual high SDS concentration (0.12%), possibly reflecting a compact overall proteasome structure.

### pH and temperature effects on CT-, T- and PGPH-like activities of 26S proteasome

**Figure 4:**
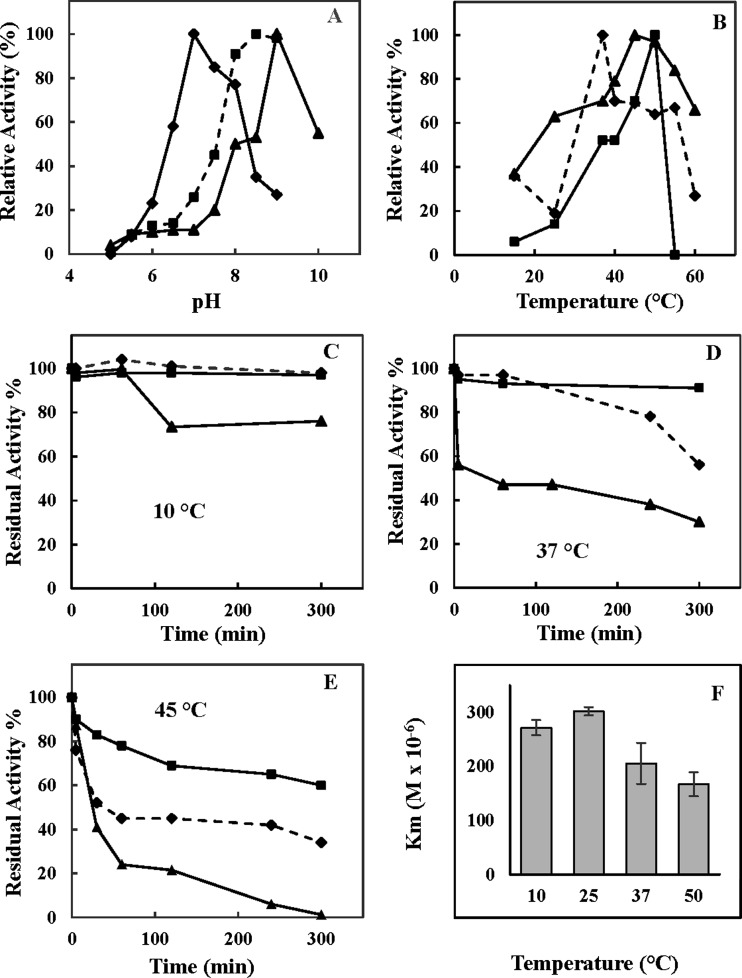
Molecular properties of the purified *T. bernacchii* 26S proteasome (**A**) pH and (**B**) temperature effects on the proteasome activities. Relative activity was expressed as percentage of the corresponding maximal activities. (**C–E**) thermoresistance of 26S isoform at: (**C**) 10°C, (**D**) 37°C, (**E**) 45°C; (**F**) temperature–*K*_m_ profile of *T. bernacchii* 26S proteasome, using LLVY as substrate. T-like, CT-like and PGPH-like activities are indicated by square, triangle and diamond respectively. All experiments were performed in triplicate on three different protein preparations. Data were expressed as means ± standard deviations. Standard deviation values lower than 5% were not shown

In studies on the effects of pH on the hydrolysing activities, we found that all the synthetic peptides used were maximally degraded at neutral and weakly alkaline pH values (Figure 4A). Specifically, the degradation of LLVY (the substrate for CT-like activity) was optimal at pH 9.0, whereas the complex had the highest activity at pH 8.5 and 7.0 towards LRR (the substrate for T-like activity) and LLE (the substrate for PGPH-like activity) respectively. All the activities gradually decreased with lowering of pH, reaching about 10% of their optima at pH values close to 5.5. The observed pH-activity profiles might be attributed to a change in ionic environment of the active sites and to a reduced access of the substrates to these sites.

To further characterize the piscine proteasome, we examined the effects of temperature on its enzymatic activities. As shown in Figure 4(B), LLVY, LRR and LLE hydrolysing activities were maximal at 45, 50 and 37°C respectively. These temperatures remained much above the optimal temperature for *T. bernacchii*, as it has also been observed for other proteases and enzymes from psychrophilic organisms [47,48]. This may suggest that the *in vitro* analyses do not reproduce the physiological conditions as several factors may greatly affect the stabilization of the protein and the enzyme–substrate recognition in cells.

Next, we explored the thermostability at 10, 37 and 45°C of the purified 26S proteasome (Figures 4C–4E). A significant different thermal behaviour was observed for the three peptidase activities only at temperatures above 10°C. Specifically, although the degradation of LLVY strongly decreased after 5 min of incubation at 37°C, LLE and LRR activities exhibited higher stabilities. At 45°C, all of them showed a gradual decline and a total inactivation was achieved for CT-like activity after 300 min of incubation.

Finally, the temperature-dependence of the Michaelis–Menten affinity constant (*K*_m_), using LLVY as substrate, was investigated. As shown in Figure 4(F), the *K*_m_ of proteasome was relatively unaffected in the temperature range 10–50°C, with the better values measured at the highest temperatures. This behaviour is in contrast with that observed with the typical cold-adapted enzymes [2,47], thus suggesting an incomplete adaptation to cold environments for the 26S complex.

### Degradation of oxidized BSA by the 26S proteasome

The production of free radicals and the consequential oxidative alteration of cell structures are ubiquitous in mammalian cells [49]. This phenomenon yields the irreversible oxidation of proteins leading to disruption of their structure and consequently to an impairment of their function. Consequently, it is necessary to remove these species in order to prevent severe metabolic disorders [49]. It is widely reported that the 20S proteasome represents the primary proteolytic system responsible for the degradation of damage oxidized proteins [13,25]. Indeed, in response to oxidative conditions or upon exposure to oxidants, the 26S holoenzyme disassembles into 20S core and 19S regulatory particles, thus favouring the 20S-mediated degradation of oxidized proteins that are not poly-ubiquitylated [16]. In contrast, there are not clear evidences about the direct implication of eukaryal 26S holoenzyme in antioxidant defense systems [24–26]. To better clarify the physiological role of 26S proteasome in Antarctic fish inhabiting under permanently oxidative stress conditions, we examined the oxidized protein hydrolase activity of this complex in *T. bernacchii.* To this aim, we raised two important questions: (*i*) whether the piscine 26S proteasome was susceptible to inactivation by oxidants, as reported in mammalian cells by Reinheckel et al. [40]; (*ii*) whether acclimation to cold temperatures could have affected the proteasome activity towards oxidized proteins.

**Figure 5:**
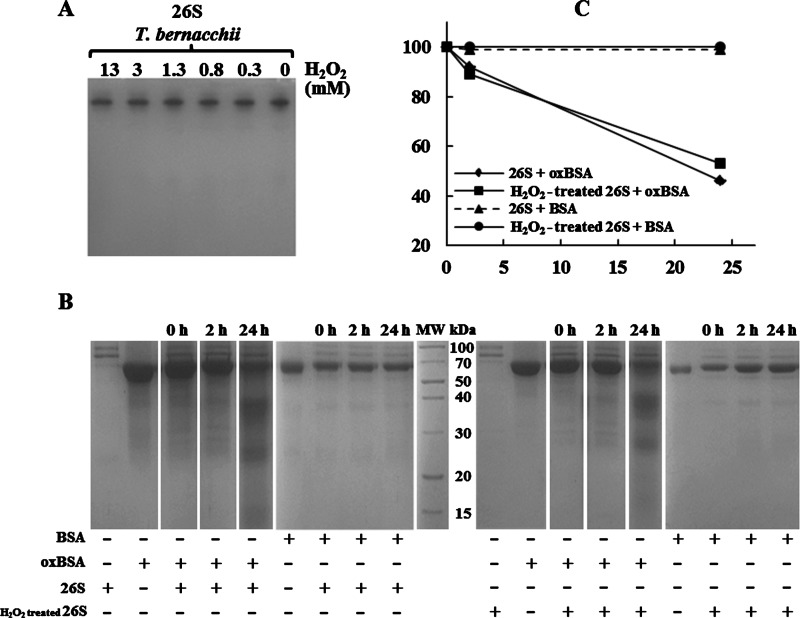
Effects of H_2_O_2_ exposure on the *T. bernacchii* 26S proteasome (**A**) Native-PAGE of *T. bernacchii* 26S proteasome complex after treatment with H_2_O_2_. Purified 26S proteasome was exposed to increasing H_2_O_2_ concentrations for 24 h at 37°C and the bands were visualized by Coomassie-blue stained. The experimental conditions for H_2_O_2_ treatment and native-PAGE are described in Materials and Methods section. (**B, *left panel***) Unoxidized and oxidized BSA were incubated with 26S proteasome at 37°C for the indicated periods; (**B, *right panel***) Unoxidized and oxidized BSA were incubated with H_2_O_2_-treated 26S proteasome at 37°C for the indicated periods. All the reaction mixtures were subjected to SDS/PAGE. (**C**) SDS/PAGE data are expressed as percentage density of BSA at the indicated incubation times compared with time 0, and were obtained by densitometric analysis with ChemiDoc XRS and Quantity One software. Oxidized BSA levels after incubation with 26S and H_2_O_2_-treated 26S are indicated by diamonds and squares respectively. Unoxidized BSA incubated with 26S and H_2_O_2_-treated 26S are indicated by triangles and circles respectively. The experiments were performed in duplicate on two different protein preparations, and the average of the relative intensities of measurements, performed in triplicate, are expressed as means ± standard deviations.

Firstly, to test the resistance of the 26S proteasome to oxidants, we exposed the isolated complex to different H_2_O_2_ concentrations (ranging from 0.3 to 150 mM) for 24 h at 37°C (Figure 5A). Surprisingly, even at high concentrations of H_2_O_2_ used, the piscine 26S proteasome did not disassemble and remained active, in contrast with that reported for the mammalian counterparts [16,26], suggesting that the individual subunits and the overall proteasome structure in fish are more resistant and less susceptible to the damaging impact of H_2_O_2_ (Figure 5A). In addition, to explore the role of the piscine 26S proteasome in response to the oxidative stress and consequently in the degradation of oxidized proteins, we carried out an experimental assay using as model substrate the BSA, a globular protein whose structure can be significantly modified by oxidative treatment [20,31]. Untreated and H_2_O_2_-treated 26S proteasome were incubated at different times with BSA, previously submitted to oxidative treatment. The unoxidized BSA was used as negative control and the protein degradation was evaluated by SDS/PAGE analysis. As shown in Figure 5(B), the intensity of the oxidized BSA band decreased remarkably after 24 h incubation with the concomitant detection of lower molecular mass fragments, following the treatment with both the unoxidized **(*left panel*)** and oxidized **(*right panel*)** 26S form. In contrast, when unoxidized BSA was used as substrate, no fragments were detected following the incubations. The obtained results were further confirmed by densitometric analysis of BSA electrophoretic bands (Figure 5C). It has been suggested that the susceptibility of oxidized BSA to proteasome degradation could be attributable to a conformational change that brings the cleavage sites, normally not exposed on the BSA surface, to the outer face of the molecule, where they become easily accessible to the enzyme upon oxidation [50]. Therefore, the hydrophobicity resulting from oxidation may represent a recognition signal for the degradation of oxidized proteins by piscine 26S proteasome.

Our data represent the first evidence of a direct involvement of the 26S proteasome in the degradation of oxidized proteins and in the antioxidant defense systems in fish inhabiting permanently cold marine environments. Therefore, the cold-adaptation in *T. bernacchii* to a highly oxidative environments may have had a greater effect on the 26S antioxidant capacity, making it more stable, less subject to disassembly and especially able to degrade oxidized proteins, differently from the isoform purified from mammalian cells [16,40]. However, further investigations are needed for a better understanding of the piscine 26S proteasome-catalysed degradation pathways.

### Cloning of the cDNAs of *T. bernacchii* 20S proteasome subunits

The rising number of sequencing projects of transcriptomes and genomes of teleosts has enriched the gene databases with sequence information of the fish proteasome subunits. However, the characterization of these sequences, mostly concerning the phylogenetic or expression analyses, has been scarce up to now [51–58]. Therefore, to gain insights into the molecular structure and evolution of the fish proteasomes, we decided to investigate the structure-function relationship of 20S proteasome from *T. bernacchii*, an organism naturally adapted to conditions of low temperature and high oxygen concentration. Specifically, based on the 3D structures of mammalian 20S [59–61], the three catalytic subunits (beta 1, beta 2 and beta 5) and those in close contact with them (alpha 4, alpha 5, alpha 7 and beta 3) were analysed. Starting from total RNA of *T. bernacchii*, the cDNAs of the seven aforementioned proteasome subunits, containing the entire coding regions, were amplified by RT-PCR using the oligonucleotides listed in Table 1 and the conditions described in Materials and Methods. The cDNAs, their predicted proteins and the relative accession numbers are listed in Table 2.

**Table 2:** cDNAs and the corresponding proteins of *T. bernacchii* proteasome subunits analysed in the present study, with relative nucleotide or amino acid lengths (in brackets) and GenBank accession numbers

cDNA (nt)	Proteasome subunit (aa)	GenBank accession number
*Beta 1_Tb_*(714)	Beta 1_Tb_ (237)	KP735942
*Beta 2_Tb_*(600)	Beta 2_Tb_ (199)	KP735943
*Beta 5_Tb_*(829)	Beta 5_Tb_ (271)	KP735944
*Alpha 4_Tb_*(786)	Alpha 4_Tb_ (261)	KP735945
*Alpha 5_Tb_*(726)	Alpha 5_Tb_ (241)	KP735946
*Alpha 7_Tb_*(759)	Alpha 7_Tb_ (252)	KP735947
*Beta 3_Tb_*(618)	Beta 3_Tb_ (205)	KP735948

### Phylogenetic analysis of *T. bernacchii* 20S proteasome catalytic subunits

The MUSCLE amino acid sequence alignments of the analysed *T. bernacchii* proteasome subunits with their homologues from organisms belonging to different kingdoms and phyla, are shown in the Supplementary Figures S1–S7. A common feature of the alignments for the non-catalytic subunits (alpha 4, alpha 5, alpha 7 and beta 3) is the very high sequence conservation (identity ranging from 87 to 100%) among those from fish, mammal and amphibian and even from phylogenetically more distant organisms (the sequence identity never fells below 45%). On the contrary, the alignments of the three catalytic subunits (beta 1, beta 2 and beta 5) showed a greater sequence variability, specifically in the N- and C-terminal regions. The beta 5 alignment revealed a significant sequence specificity among the different phylogenetic groups, accompanied by a low overall degree of conservation (the minimum sequence identity value was 35% among all the species). On the contrary, the remaining two catalytic subunits, beta 1 and beta 2, displayed a greater sequence conservation among fish, mammal and amphibian (identity ranging from 77 to 99%), which strongly dropped down when the other species were considered (the minimum identity value: 14%).

**Figure 6:**
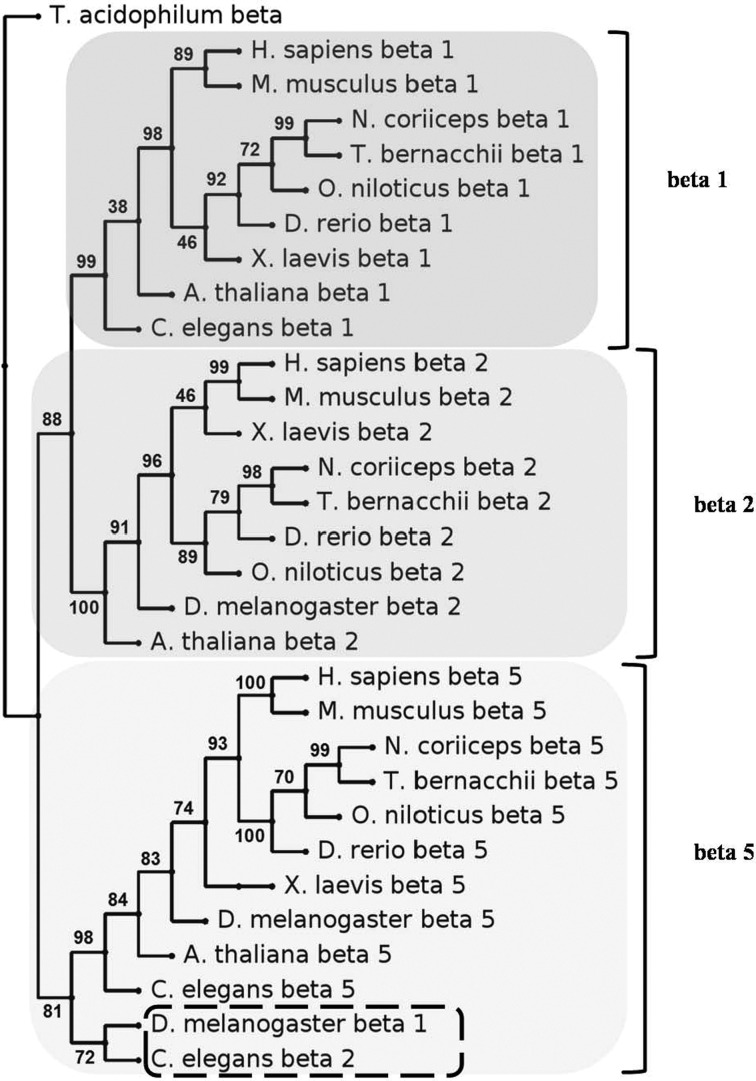
Phylogenetic analysis of the catalytic subunits of *T. bernacchii* 20S proteasome The amino acid sequences of beta 1, beta 2 and beta 5 subunits of *T. bernacchii* were compared with their respective homologues from teleosts [*D. rerio*, *O. niloticus* and *Notothenia coriiceps* (the latter is an Antarctic Nototheniide)], mammals (*Homo sapiens* and *M. musculus)*, amphibian (*X. laevis)*, insect (*D. melanogaster)*, nematode (*C. elegans)*, plant (*A. thaliana)*, whose species names and accession numbers are shown in the alignment of Supplementary Figure S8. The unique beta subunit sequence from the archaebacterium *T. acidophilum* was used as outgroup. A tree with the best bootstrap consensus is shown, with numbers at nodes representing the confidence limits computed by the bootstrap procedure (1000 replicates). The clusters relative to the three beta subfamilies are indicated.

Following the procedure described in Materials and Methods and using the sequence of the unique beta subunit of proteasome from the archaebacterium *T. acidophilum* as outgroup (Supplementary Figure S8), a phylogenetic analysis for the three catalytic subunits was carried out and the tree with the best bootstrap consensus was built (Figure 6).

As expected, distinct clusters for the three subunits were revealed, with some discrepancies in the branch order within the individual organism subfamilies, which affected the rooting position in the tree of the sequences of *D. rerio* and *O. niloticus* within fish, *Xenopus laevis* within vertebrates, *Drosophila melanogaster* and *Caenorhabditis elegans* within invertebrates and *Arabidopsis thaliana* within plants. Interestingly, the sequences of *D. melanogaster* beta 1 and *C. elegans* beta 2 clustered together with those of beta 5 subfamily, rather than with those of their respective groups. These anomalies, already described [62], represent a peculiarity within these clusters and are the result of evolutionary events involving this important enzyme complex, which make very difficult the phylogenetic analysis.

### Molecular modelling of *T. bernacchii* proteasome subunits

The availability of the 3D structures of proteasome from different sources allowed to approach a molecular modelling analysis of seven *T. bernacchii* proteasome subunits, whose cDNAs have been cloned in the present study (Table 2). Specifically, due to the high level of sequence identity (>80%) and sequence coverage (>90%) between the subunits of *T. bernacchii* and the corresponding proteins from *Mus musculus*, we chosen the 3D murine structure of 20S proteasome as template. Interestingly, the beta 1 and beta 5 subunits from *T. bernacchii* presented dissimilarities in the N-terminal region, including 24 and 68 amino acids respectively, with no reference in the corresponding murine subunits.

**Figure 7:**
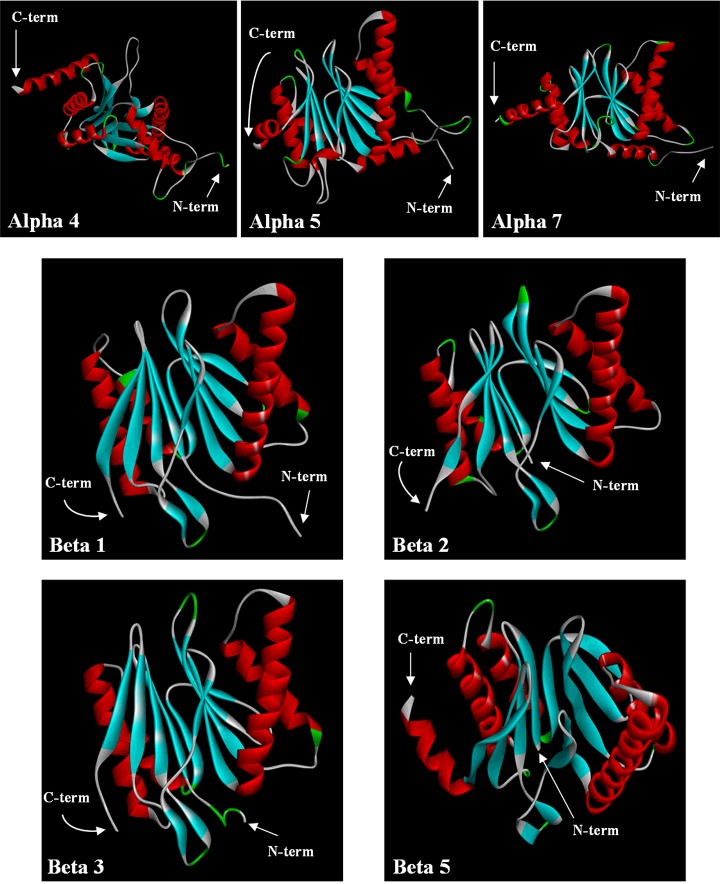
Schematic view of the backbone fold of seven proteasome subunits from *T. bernacchii* Alpha helix, beta-strand and turn structures are indicated with red, cyan and green colours respectively. The positions of N- and C-termini are indicated by arrows. Models were generated as described in Materials and Methods section. Images have been created with Discovery Studio software.

According to the modelling procedures in use by our group (see Materials and Methods for details), we generated ten 3D models for each *T. bernacchii* subunits and then we selected the best structures in terms of stereochemical properties and energy evaluation. We report the values obtained with Procheck and ProsaWeb analyses in Supplementary Table S2. The obtained results clearly indicate a high quality and in some cases better energy parameters of the built models respect to the structures of the murine template, suggesting that the piscine subunits are well suited into their respective known fold. The 3D models of the seven subunits under investigation are reported in Figure 7. In addition, as shown in Table 3, a comparative analysis between *T. bernacchii* and mouse subunits was carried out, evaluating the number of some stabilizing factors, such as H-bonds, salt-bridges and cysteine residues. Specifically, although the increased H-bonds numbers suggested a higher stability for all the *T. bernacchii* subunits (other than the beta 1) respect to the corresponding mouse counterparts, a clear predominance in the number of the intra-chain salt-bridges between the proteasome subunits of the two organisms was not observed (Table 3). On the other hand the cysteine residues, whose presence might suggest the formation of intra- or inter-chain disulfide bonds, are well conserved in the murine and piscine subunits, excepted for the beta 2 (the same total number is due to a couple of aligned cysteine residues and another one presents in different positions) and the beta 5 subunits (Table 3). Specifically, the *T. bernacchii* beta 5 has nine cysteine residues against the three in mouse. Four of them are in the N-terminal region of 68 amino acids that, as mentioned, is not included in the 3D model of this subunit, whereas the remaining five cysteine residues could be involved in the formation of inter-chains bonds, being not adjacent in the space.

**Table 3:** H-bonds, intra-chain salt bridges and cysteine residues in the proteasome subunits from *T. bernacchii* and *M. musculus*

	H-bonds		Intra-chain salt bridges		Cysteine residues	
	*T. bernacchii*	*M. musculus*	*T. bernacchii*	*M. musculus*	*T. bernacchii*	*M. musculus*
Alpha 4	208	204	10	11	5	5
Alpha 5	181	176	8	7	3	3
Alpha 7	162	157	12	15	3	3
Beta 1	169	177	9	8	4	4
Beta 2	158	151	10	8	3	3
Beta 3	159	153	5	5	5	5
Beta 5	176	172	6	7	9	3

These analyses have been focused on the single modelled subunits, which are expected to be near in the quaternary structure of the piscine proteasome by similarity to the corresponding murine isoform; however, for illustrative purposes, we generated a putative assembly for *T. bernacchii* 20S proteasome, in order to have an idea on the spatial arrangements of the subunits under investigation within the protein complex (Supplementary Figure S9).

Nevertheless, the ability to create inter-chain salt-bridges, that could modulate the flexibility of the entire proteasome assembly, can be investigated by considering what inter-chain interactions are present in the murine proteasome, and verifying the conservation in *T. bernacchii* subunits of the involved residues. In Table 4 are reported the number of inter-chain salt-bridges observed in mouse proteasome for the chains of interest. Interestingly, all the amino acids involved in the murine proteasome salt-bridges are well conserved between the two species and they are all present in *T. bernacchii*, except for two residues of beta 5 chain, a Serine and a Glutamine, which are the Arg^141^ and the Asp^197^ in the corresponding subunit in mouse. Therefore, the *T. bernacchii* catalytic subunit beta 5 lacks the possibility to create two out of 16 inter-chain salt-bridges observed in the murine proteasome structure, with possible effects on the ability of the entire proteasome to adapt to substrates.

**Table 4:** Analysis of inter-chain salt bridges in murine proteasome involving the seven subunits of interest

	Number of salt-bridges	Amino acids involved
Alpha 4	8	Arg^2^, Arg^7^, Asp^56^, Asp^115^, Glu^107^, Glu^180^
Alpha 5	12	Asp^1^, Glu^52^, Glu^61^, Asp^63^, Asp^82^, Glu^102^, Glu^117^, Glu^118^, Asp^119^, Glu^140^
Alpha 7	9	Arg^4^, Arg^35^, Arg^56^, Arg^80^, Glu^98^, Glu^99^, Asp^138^, Lys^156^
Beta 1	8	Asp^60^, Lys^66^, Lys^81^, Arg^100^, Asp^150^, Glu^184^, Arg^185^, Asp^213^
Beta 2	8	Glu^49^, Glu^58^, Asp^90^, Arg^93^, Asp^144^, Glu^165^, Glu^166^, Arg^170^
Beta 3	14	Asp^58^, Arg^65^, Arg^79^, Arg^98^, Glu^150^, Glu^154^, Glu^160^, Arg^176^, Arg^197^, Lys^200^, Asp^204^
Beta 5	16	Arg^19^, Asp^51^, Arg^64^, Arg^69^, Lys^81^, Lys^91^, Lys^106^, Arg^107^, Arg^120^, Asp^140^, Arg^141^, Arg^166^, Asp^197^

## CONCLUSIONS

One of the most important physical effects related to the lowering temperature is the increase in the oxygen solubility in water. For this reason, the Antarctic marine environment represents a unique natural habitat that deeply affects the life of its organisms [17,18]. Specifically, the Antarctic fish have had to adopt alternative strategies essential for their survival, being permanently subjected to a higher oxidative stress than the temperate species [1,3–6]. Therefore, these organisms represent a fascinating model to study the biological processes involved in protein degradation and antioxidant defense mechanisms.

In such a context, the purpose of this work was to shed important light on understanding such processes in organisms adapted to living under cold-induced oxidative stress conditions, through the investigation on the main enzyme system involved in the degradation pathway of damaged and oxidized proteins: the proteasome. To this aim, the Antarctic notothenioid *T. bernacchii* was used as biological model system.

The present study highlighted two significant findings: 1) the protein degradation machinery in the cold-adapted specie seems to be more efficient respect to that of the temperate fish. 2) The cold-adaptation in *T. bernacchii* may have had a greater effect on the 26S antioxidant capacity, making it more stable, less subject to disassembly and especially able to degrade oxidized proteins, differently from the isoform purified from mammalian cells [16,40]. These unique functional properties were also reflected by the analysis of the 3D models of seven proteasome subunits, which revealed a higher structural stability of the piscine complex respect to the murine template.

Further investigations are needed in order to determine if the oxidative-resistance and the uncommon properties displayed by the *T. bernacchii* proteasome are the result of a cold adaptation or an intrinsic feature of the piscine isoforms.

## Abbreviations

BSA: bovine serum albumin
CP: proteasome core particle
CT-like: chymotrypsin-like activity
LLE: *tert*-butyloxycarbonyl-Leu-Leu-Glu-7-amido-4-methylcoumarin
LLVY: *N*-succinyl-Leu-Leu-Val-Tyr-7-amido-4-methylcoumarin
LRR: *tert*-butyloxycarbonyl-Leu-Arg-Arg-7-amido-4-methylcoumarin
PGPH-like: caspase-like activity
T-like: trypsin-like activity
UPS: ubiquitin–proteasome system

## References

[1] Chen L., DeVries A.L., Cheng C.-H.C. (1997). Evolution of antifreeze glycoprotein gene from a trypsinogen gene in Antarctic notothenioid fish. Proc. Natl. Acad. Sci. U.S.A..

[2] Somero G.N. (2004). Adaptation of enzymes to temperature: searching for basic “strategies”. Comp. Biochem. Physiol. B Biochem. Mol. Biol..

[3] Cheng C.-H.C., Detrich H.W. (2007). Molecular ecophysiology of Antarctic notothenioid fishes. Phil. Trans. R. Soc. B.

[4] O'Brien K.M., Mueller I.A. (2010). The unique mitochondrial form and function of antarctic Channichthyid icefishes. Integr. Comp. Biol..

[5] Lamarre S.G., Blier P.U., Driedzic W.R., Le Francois N.R. (2010). White muscle 20S proteasome activity is negatively correlated to growth rate at low temperature in the spotted wolffish *Anarhichas minor*. J. Fish. Biol..

[6] Hofmann G.E., Buckley B.A., Airaksinen S., Keen J.E., Somero G.N. (2000). Heat shock protein expression is absent in the antarctic fish *Trematomus bernacchii* (family Nototheniidae). J. Exp. Biol..

[7] Jaenicke R. (1990). Protein structure and function at low temperature. Philos. Trans. R. Soc. Lond. B Biol. Sci..

[8] Jaenicke R. (1991). Protein stability and molecular adaptation to extreme conditions. Eur. J. Biochem..

[9] Fujita J. (1999). Cold shock response in mammalian cells. J. Mol. Microbiol. Biotechnol..

[10] Todgham A.E., Hoaglund E.A., Hofmann G.E. (2007). Is cold the new hot? Elevated ubiquitin-conjugated protein levels in tissues of Antarctic fish as evidence for cold-denaturation of proteins *in vivo*. J. Comp. Physiol. B.

[11] Hochstrasser M. (1995). Ubiquitin, proteasomes, and the regulation of intracellular protein degradation. Curr. Opin. Cell Biol..

[12] Lecker S.H., Goldberg A.L., Mitch W.E. (2006). Protein degradation by the ubiquitin–proteasome pathway in normal and disease states. J. Am. Soc. Nephrol..

[13] Sorokin A.V., Kim E.R., Ovchinnikov L.P. (2009). Proteasome system of protein degradation and processing. Biochemistry (Mosc).

[14] Kim H.M., Yu Y., Cheng Y. (2011). Structure characterization of the 26S proteasome. Biochim. Biophys. Acta.

[15] da Fonseca P.C.A., He J., Morris E.P. (2012). Molecular model of the human 26S proteasome. Mol. Cell.

[16] Livnat-Levanon N., Kevei E., Kleifeld O., Krutauz D., Segref A., Rinaldi T., Erpapazoglou Z., Cohen M., Reis N., Hoppe T., Glickman M.H. (2014). Reversible 26S proteasome disassembly upon mitochondrial stress. Cell Rep..

[17] Regoli F., Benedetti M., Krell A., Abele D., Abele D., Vazquez-Medina J.P., Zenteno-Savin T. (2012). Oxidative challenges in polar seas. Oxidative stress in aquatic ecosystems.

[18] Eastman J.T. (2004). The nature of the diversity of Antarctic fishes. Polar Biol..

[19] Abele D., Puntarulo S. (2004). Formation of reactive species and induction of antioxidant defence systems in polar and temperate marine invertebrates and fish. Comp. Biochem. Physiol. A Mol. Integr. Physiol..

[20] Gogliettino M., Riccio A., Balestrieri M., Cocca E., Facchiano A., D'Arco T.M., Tesoro C., Rossi M., Palmieri G. (2013). A novel class of bifunctional acylpeptide hydrolases: potential role in the antioxidant defense systems of the Antarctic fish *Trematomus bernacchii*. FEBS J..

[21] Riccio A., Gogliettino M., Palmieri G., Balestrieri M., Facchiano A., Rossi M., Palumbo S., Monti G., Cocca E. (2015). A new APEH cluster with antioxidant functions in the antarctic hemoglobinless icefish *Chionodraco hamatus*. PLoS One.

[22] Benedetti M., Nigro M., Regoli F. (2010). Characterisation of antioxidant defences in three Antarctic notothenioid species from Terra Nova Bay (Ross Sea). Chem. Ecol..

[23] Ben-Nissan G., Sharon M. (2014). Regulating the 20S proteasome ubiquitin-independent degradation pathway. Biomolecules.

[24] Höhn T.J., Grune T. (2014). The proteasome and the degradation of oxidized proteins: Part III-redox regulation of the proteasomal system. Redox Biol..

[25] Davies K.J.A. (2001). Degradation of oxidized proteins by the 20S proteasome. Biochimie.

[26] Aiken C.T., Kaake R.M., Wang X., Huang L. (2011). Oxidative stress-mediated regulation of proteasome complexes. Mol. Cell. Proteomics.

[27] Bradford M.M. (1976). A rapid and sensitive method for the quantitation of microgram quantities of protein utilizing the principle of protein-dye binding. Anal. Biochem..

[28] Laemmli U.K. (1970). Cleavage of structural proteins during the assembly of the head of bacteriophage T4. Nature.

[29] Holzl H., Kapelari B., Kellermann J., Seemuller E., Sumegi M., Udvardy A., Medalia O., Sperling J., Muller S.A., Engel A., Baumeister W. (2000). The regulatory complex of *Drosophila melanogaster* 26S proteasomes: Subunit composition and localization of a deubiquitylating enzyme. J. Cell Biol..

[30] Leggett D.S., Glickman M.H., Finley D. (2005). Purification of proteasomes, proteasome subcomplexes, and proteasome-associated proteins from budding yeast. Methods Mol. Biol..

[31] Fujino T., Ishikawa T., Inoue M., Beppu M., Kikugawa K. (1998). Characterization of membrane-bound serine protease related to degradation of oxidatively damaged erythrocyte membrane proteins. Biochim. Biophys. Acta.

[32] Tamura K., Stecher G., Peterson D., Filipski A., Kumar S. (2013). MEGA6: molecular evolutionary genetics analysis version 6.0. Mol. Biol. Evol..

[33] Marabotti A., Facchiano A.M. (2005). Homology modeling studies on human galactose-1-phosphate uridylyltransferase and on its galactosemia-related mutant Q188R provide an explanation of molecular effects of the mutation on homo-and heterodimers. J. Med. Chem..

[34] http://salilab.org/modeller/.

[35] Wiederstein M., Sippl M.J. (2007). ProSA-web: interactive web service for the recognition of errors in three-dimensional structures of proteins. Nucleic Acid Res..

[36] Laskowski R.A., MacArthur M.W., Moss D.S., Thornton J.M. (1993). PROCHECK: a program to check the stereochemical quality of protein structures. J. Appl. Cryst..

[37] McDonald I.K., Thornton J.M. (1994). Satisfying hydrogen bonding potential in proteins. J. Mol. Biol..

[38] Paladino A., Costantini S., Colonna G., Facchiano A.M. (2008). Molecular modelling of miraculin: structural analyses and functional hypotheses. Biochem. Biophys. Res. Commun..

[39] Tai H.C., Besche H., Goldberg A.L., Schuman E.M. (2010). Characterization of the brain 26S proteasome and its interacting proteins. Front. Mol. Neurosci..

[40] Reinheckel T., Sitte N., Ullrich O., Kuckelkorn U., Davies K.J., Grune T. (1998). Comparative resistance of the 20S and 26S proteasome to oxidative stress. Biochem. J..

[41] Bousquet-Dubouch M.P., Baudelet E., Guérin F., Matondo M., Uttenweiler-Joseph S., Burlet-Schiltz O., Monsarrat B. (2009). Affinity purification strategy to capture human endogenous proteasome complexes diversity and to identify proteasome-interacting proteins. Mol. Cell. Proteomics.

[42] Bajorek M., Glickman M.H. (2004). Keepers at the final gates: regulatory complexes and gating of the proteasome channel. Cell. Mol. Life Sci..

[43] Forster A., Whitby F.G., Hill C.P. (2003). The pore of activated 20S proteasomes has an ordered 7-fold symmetric conformation. EMBO J..

[44] Orlowski M., Wilk S. (2003). Ubiquitin-independent proteolytic functions of the proteasome. Arch. Biochem. Biophys..

[45] Ugai S., Tamura T., Tanahashi N., Takai S., Komi N., Chung C.H., Tanaka K., Ichihara A. (1993). Purification and characterization of the 26S proteasome complex catalyzing ATP-dependent breakdown of ubiquitin-ligated proteins from rat liver. J. Biochem..

[46] Dutaud D., Aubry L., Sentandreu M.A., Ouali A. (2006). Bovine muscle 20S proteasome: I. Simple purification procedure and enzymatic characterization in relation with postmortem conditions. Meat Sci..

[47] Brunialti E.A., Gatti-Lafranconi P., Lotti M. (2011). Promiscuity, stability and cold adaptation of a newly isolated acylaminoacyl peptidase. Biochimie.

[48] Parravicini F., Natalello A., Papaleo E., De Gioia L., Doglia S.M., Lotti M., Brocca S. (2013). Reciprocal influence of protein domains in the cold-adapted acyl aminoacyl peptidase from *Sporosarcina psychrophila*. PLoS One.

[49] Kehrer J.P., Robertson J.D., Smith C.V., McQueen C. (2010). Free radicals and reactive oxygen species. Comprehensive Toxicology.

[50] Fujino T., Kojima M., Beppu M., Kikugawa K., Yasuda H., Takahashi K. (2000). Identification of the cleavage sites of oxidized protein that are susceptible to oxidized protein hydrolase (OPH) in the primary and tertiary structures of the protein. J. Biochem..

[51] Tokumoto M., Horiguchi R., Nagahama Y., Ishikawa K., Tokumoto T. (2000). Two proteins, a goldfish 20S proteasome subunit and the protein interacting with 26S proteasome, change in the meiotic cell cycle. Eur. J. Biochem..

[52] Clark M.S., Pontarotti P., Gilles A., Kelly A., Elgar G. (2000). Identification and characterization of a beta proteasome subunit cluster in the Japanese pufferfish (*Fugu rubripes*). J. Immunol..

[53] Tsukamoto K., Miura F., Fujito N.T., Yoshizaki G., Nonaka M. (2012). Long-lived dichotomous lineages of the proteasome subunit beta type 8 (PSMB8) gene surviving more than 500 million years as alleles or paralogs. Mol. Biol. Evol..

[54] Sutoh Y., Kondo M., Ohta Y., Ota T., Tomaru U., Flajnik M.F., Kasahara M. (2012). Comparative genomic analysis of the proteasome β5t subunit gene: implications for the origin and evolution of thymoproteasomes. Immunogenetics.

[55] Kasthuri S.R., Umasuthan N., Whang I., Kim E., Park H.C., Lee J. (2013). Genomic structural characterization and transcriptional expression analysis of proteasome activator PA28α and PA28β subunits from *Oplegnathus fasciatus*. Fish Shellfish Immunol..

[56] Kasthuri S.R., Umasuthan N., Whang I., Lim B.S., Jung H.B., Oh M.J., Jung S.J., Yeo S.Y., Kim S.Y., Lee J. (2014). Molecular characterization and expressional affirmation of the beta proteasome subunit cluster in rock bream immune defense. Mol. Biol. Rep..

[57] Salmerón C., Navarro I., Johnston I.A., Gutiérrez J., Capilla E. (2015). Characterisation and expression analysis of cathepsins and ubiquitin-proteasome genes in gilthead sea bream (*Sparus aurata*) skeletal muscle. BMC Res. Notes.

[58] Rolland M., Dalsgaard J., Holm J., Gómez-Requeni P., Skov P.V. (2015). Dietary methionine level affects growth performance and hepatic gene expression of GH-IGF system and protein turnover regulators in rainbow trout (*Oncorhynchus mykiss*) fed plant protein-based diets. Comp. Biochem. Physiol. B Biochem. Mol. Biol..

[59] Unno M., Mizushima T., Morimoto Y., Tomisugi Y., Tanaka K., Yasuoka N., Tsukihara T. (2002). The structure of the mammalian 20S proteasome at 2.75 A resolution. Structure.

[60] da Fonseca P.C., Morris E.P. (2008). Structure of the human 26S proteasome: subunit radial displacements open the gate into the proteolytic core. J. Biol. Chem..

[61] Huber E.M., Basler M., Schwab R., Heinemeyer W., Kirk C.J., Groettrup M., Groll M. (2012). Immuno- and constitutive proteasome crystal structures reveal differences in substrate and inhibitor specificity. Cell.

[62] Volker C., Lupas A.N. (2002). Molecular evolution of proteasomes. Curr. Top. Microbiol. Immunol..

